# Comparing time and motion methods to study personnel time in the context of a family planning supply chain intervention in Senegal

**DOI:** 10.1186/s12960-018-0328-2

**Published:** 2018-11-19

**Authors:** Elizabeth McElwee, Jenny A. Cresswell, Christian Yao, Macaire Bakeu, Francesca L. Cavallaro, Diane Duclos, Caroline A. Lynch, Lucy Paintain

**Affiliations:** 1 London School of Hygiene & Tropical Medicine, London, United Kingdom; 2 CSD Convergence Santé pour le Développement, Dakar, Senegal; 3 Centre Africain d’études Supérieures en Gestion, Dakar, Senegal; 4 Washington, DC, United States of America

**Keywords:** Time and motion study, Supply chain, Labour (labor) costs, Senegal, Informed push model, Continuous observation, Self-administered timesheets

## Abstract

**Background:**

A family planning (FP) supply chain intervention was introduced in Senegal in 2012 to reduce contraceptive stock-outs. Labour is the highest cost in low- and middle-income country supply chains. In this paper, we (1) understand time use of personnel working in the FP supply chain at health facilities in Senegal, (2) estimate the validity of self-administered timesheets (STs) relative to continuous observations (COs), and (3) describe the cost of data collection for each method.

**Methods:**

We collected time use data for seven stockroom managers in six facilities using both ST and CO. Activities were categorized as follows: stock management associated with FP, non-FP stock management, other productive activities, non-productive activities, and waiting time. Paired *t* tests were used to compare the mean differences between the two methods in all categories and in productive time alone.

**Results:**

Among all activities, the absolute and relative time spent on productive activities was higher when estimated by ST compared to CO. Conversely, waiting time was underestimated by STs. There was no difference in the relative time spent on non-productive activities. When comparing the distribution of the three productive activity categories, we found no evidence of a difference in relative time percentage estimates between CO and ST (FP stockroom management − 3.0%, 95% CI − 7.4 to 1.4%; non-FP stockroom management 3.4%, 95% CI − 2.8 to 9.6%; and other productive activities − 0.1%, 95% CI − 6.3 to 6.0%). Data collection costs for CO are 140% more than ST.

**Conclusion:**

STs were not a reliable method for measuring absolute labour time at health facilities in Senegal due to considerable underestimates of time waiting for clients. However, ST had acceptable reliability when examining distribution of productive time. Although CO provides more accurate absolute time estimates, the unit costs for data collection using this method are more than triple those for STs in Senegal.

## Key messages

- Stockroom managers overestimate the absolute and relative time spent on productive tasks and underestimate waiting time when filling out a self-administered timesheet (ST) as compared to results of continuous observations (COs).
- For productive tasks only, STs are a reliable and affordable time and motion study method to measure relative time stock room managers spend on tasks in Senegal, as compared with COs.
- Future studies that plan to calculate personnel time should incorporate a pilot study, to compare differences in CO and ST, and understand the difference in mean times collected by the two methods to appropriately weigh any results for a given context.

## Background

In 2012, the Senegalese Ministry of Health sought to reduce unmet need for family planning (FP) by addressing stock-outs of contraceptives with an FP supply chain intervention called the Informed Push Model with third party logisticians (IPM-3PL) [1, 2]. Under the IPM-3PL supply chain, deliveries of FP commodities are made directly from the regional level to the service delivery points—bypassing the district stockrooms—by a private operator contracted through an international non-governmental organization. One objective of IPM-3PL was to reduce the time health workers at the service delivery points needed to spend on stock-taking activities, giving them more time to deliver FP services [1, 2]. Health workers’ time is one of the costliest components of low- and middle-income countries’ (LMICs) supply chains [3–5] and FP service provision [6]. Thus, it is necessary to understand all costs involved in supply chain interventions, like IPM-3PL, to ensure resources available in Senegal are allocated efficiently. To assess the costs involved in this FP supply chain, identification of reliable and affordable time and motion study (TMS) methods to collect personnel time costs is needed.

It is challenging to calculate personnel time costs associated with supply chains in low-income settings. Data on health workers’ time, salaries, and supply inventory are not readily available, since electronic stock management databases are rare at the service delivery points and salaries are frequently paid by various sources [7–11]. An alternative way to capture time spent on different activities in healthcare settings is with a TMS which can involve many different methods, utilizing either an external observer or self-reporting [12]. In order to find appropriate time and motion methods to calculate health workers’ time costs, a literature review was conducted to find all publications that discussed time and motion studies in a healthcare setting. We first searched the PubMed database using the terms “personnel costs”, “health workers’ time”, “time and motion methods/studies”, continuous observations, and self-administered time sheets. We then reviewed the reference sections of identified articles to find additional literature not found in our initial search.

Methods using external observers include continuous observation (CO) where an observer documents the activities of one person for a whole shift and records each activity separately including the start and end times [12, 13]; work sampling where an observer records activities performed at set time intervals [14, 15]; or patient flow analysis where each patient is given a form to track all activities as they flow through a health clinic, from entering to leaving [7]. Methods for self-reporting include provider interviews (PIs) which are undertaken at one point in time with personnel who are asked to describe a typical day including what proportion of their time is spent on different activities [7, 16]; personal diaries in which staff record their perceptions of how they spend their time over predetermined time periods [10]; or self-administered timesheets (ST) where personnel fill out a timesheet referencing each task performed in a shift, either while working or at the end of the shift [7, 8, 17, 18].

In high-resource healthcare settings, COs are accepted as the preferred method to collect time/duration data [12]. Comparisons of CO and ST have been carried out in two high-resource settings, Australia and the United States of America. In the Australian study, CO and STs were carried out with nine nurses and they concluded that CO is a better method because the nurses had little time to fill out the STs, leading to only a 56% compliance rate for the STs. These nurses tended to under-report patient care and over-report time spent on documentation [7, 16]. The study in the United States of America observed eight nurses over five shifts and then had them complete STs for the next five shifts. This study recognized that self-reporting of time can be a low-cost way to quantify how personnel use their time, specifically the relative time spent on specific tasks. However, they stress that STs are less valid for reporting the absolute time it takes to complete a task or in estimating the total number of activities a health worker completes on a daily basis [17].

A limited number of studies exist that have compared one or more TMS methods in low-resource healthcare settings; we are not aware of other studies that specifically target workers involved in supply chain logistics or stockroom management in LMICs. Below, we include all TMS studies in the healthcare setting and TMS method comparison studies that we found for LMICs. One comparison study in Ecuador, which is frequently cited for TMS methods in LMICs, used the a priori assumption that CO is the gold standard. In this study, Bratt et al. used TMS to examine patient flow for clinicians in reproductive health services at the health facility level and concluded that CO was the most reliable technique to quantify health workers’ time as compared to PIs, STs, and patient flow analysis. STs were also found to be a valid indicator of time spent on specific, productive tasks but underestimated non-productive time as compared to CO [7]. The study was conducted in three clinics in Ecuador, so the extent to which the findings are generalizable to other contexts is unknown, but it did provide guidelines for reporting results in a TMS methods comparison study, and subsequent time and motion studies have since used this research to claim CO as the “gold standard” of TMS methods in LMICs. Another study in Tanzania compared in-depth PIs with 1 week of COs and found that health workers underestimated their unproductive time in the interviews and that conducting the interviews before the observations allowed them to reduce the Hawthorne effect [8]. Ultimately, this study used the interviews as a complement to the CO and did not fully compare the methods.

We conducted a TMS comparison study of stockroom managers working with supply chains at service delivery points in Senegal in the context of the new IPM-3PL intervention. Our study had three objectives: (1) to understand time use of personnel working with the family planning supply chain at service delivery points in this setting, (2) to estimate the validity of ST relative to CO as a gold standard to quantify personnel time in the Senegalese context; and (3) to describe the cost of data collection to the research team for both ST and CO. This study was a pilot that took place within a larger evaluation of IPM-3PL throughout Senegal.

## Methods

### Study setting

In Senegal, the public health system consists of national-, regional-, district-, and local-level facilities. Medical products are supplied from national to regional level. In the non-IPM-3PL supply-chain system that is for all products except contraceptives, a stockroom manager at the district level receives and collates medical product orders from lower-level facilities and then collects these orders from the region. Lower-level facilities are responsible for ensuring transport of medical supplies from the district to their facility. As well as placing orders, the duties of a stockroom manager (*depositaire*) at lower-level health facilities include dispensing prescribed medicines, maintaining inventory of medicines, and working with the clinic managers to share the task of ordering and picking up all medicines at the district depots. Under IPM, the private operator removes the tasks of placing and picking up FP orders from the stockroom manager and shares in the responsibility of conducting the physical inventory of FP stock during monthly visits to the health facility. All stockroom managers in our study are paid by a village health committee, which are part of Senegal’s decentralized system for making health system choices. Some stockroom managers are paid with a fixed salary and some on a commission-only basis where they receive a percentage of monthly drug sales, so their income fluctuates monthly while their hours worked do not, ultimately affecting personnel time costs. An average working day for health personnel in sampled districts in Senegal is 5.5 h.

### Study design

We selected six service delivery points using purposive sampling: one health centre and two health posts in two districts from two different regions (Dakar and Thiès), both of which are relatively urban. Since Senegal has a decentralized health system, we chose to explore health centres and health posts in two different regions to explore variation among regions as well as between health centres and health posts. Health posts and health clinics were chosen based on their size, estimated by average patient volumes per month, high (> 1 000 patients), medium, and low populations (< 200 patients). All the participating health posts and one health centre had one stockroom manager; in the other health centre, there was a supervising stockroom manager and two assistants. Prior to the time and motion study, we conducted interviews with personnel at service delivery points to understand which staff members were most involved with IPM-3PL and the supply chain.

Stockroom managers were identified as the cadre most affected by the implementation of IPM-3PL, so they were selected as the health workers to include in the study. In general, we found that the midwives who provide FP services were not usually involved in day to day of IPM-3PL or supply chain logistics. Prior to the time and motion study, we interviewed stockroom managers at one health post and one health centre, which were not included in our TMS, to identify their main work activities and incorporated these activities into the self-administered timesheet design. This ST was piloted at one health facility, where two observers, who were external to the health facilities, were trained over 2 days. In assessing inter-observer reliability, the external observers’ observations were found to be within 5 min of each other for each task category, with the main difference seen in the categorization of talking with co-worker as either productive or unproductive. This difference was discussed, and conversations with co-workers were coded as a productive task. Based on testing, edits were made to the ST tool to ensure the health workers understood the time categories and the final tool was created [see Additional file 1: timesheet, PDF].

One stockroom manager underwent CO and completed the ST in each facility. However, in the health centre with three stockroom managers, the supervising stockroom manager was observed as well as one of the stockroom assistants, chosen randomly, and each was observed by a different observer. In total, seven stockroom employees were observed in our study. All study visits were carried out on weekdays from June 2015 through July 2015.

We carried out a CO in each health facility for two consecutive days, totalling 14 observation days with seven stockroom managers. The two trained observers recorded all tasks completed by the stockroom managers using log sheets and a watch, noting the start and stop time of each activity to the nearest minute [see Additional file 2 CO log sheet, PDF]. The observers were randomly assigned to a health facility, and the same observer completed the CO on both days. Observers sat in an unobtrusive area where they could see all actions of the stockroom manager; if needed, they shadowed them in other areas of the health facility. No staff turnover occurred during the study, and no stockroom managers were absent on any observation days.

At the end of each observed day, the stockroom manager filled out a two-page ST (Appendix 1). The STs were completed on the same days as the CO before the observer left to enable direct comparison with the tasks observed in the CO. The observers assisted the health worker as needed to fill out the sheet, as French was not all of the stockroom managers’ first language and the tool was in French. The observers prompted the participants in general terms to reflect on all the activities of the day; however, they did not provide any time estimates to the participants, but allowed the participants to record the times they thought appropriate.

Data collection costs reported in this paper, namely the wages of fieldworkers and the driver, and the cost of fuel according to the mileage, were collected from project financial records.

Our study protocol and supporting documents were submitted to both the LSHTM and Senegal Ministry of Health ethics committee. Both LSHTM ethics review board (reference 9925) and the Senegalese Ministry of Health (reference 15/35) gave ethical approval for this study. Permission was also obtained from the regional and district managers where the study took place. Written informed consent was obtained from all participants. All data in this study was anonymized prior to analysis.

### Data analysis

**Fig. 1:**
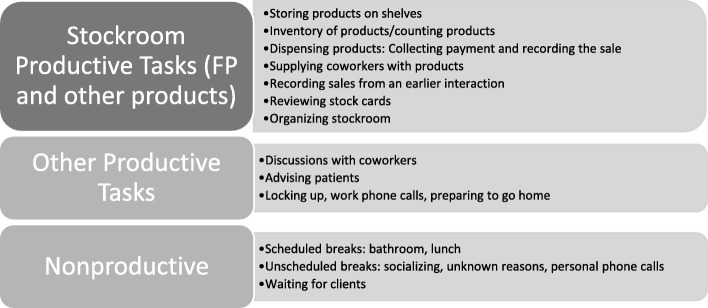
List of tasks observed in CO and reported in ST

A descriptive analysis of absolute time spent on different activity categories was calculated for both CO and ST. Tasks were grouped according to (i) productive time associated with stockroom management of FP products; (ii) productive time associated with stockroom management for all other non-FP products; (iii) productive time for all other types of activities; (iv) time waiting for patients; and (v) other non-productive time (Fig. 1). The observers recorded which medications the stockroom managers were distributing for each set of tasks; thus, we were able to distinguish family planning activities from all other activities. Relative time spent on each task was calculated by dividing the total time in each category by the total work time and presented as a percentage. We focused on relative time to adjust for different schedules of stockroom managers and to convert the time spent on tasks into a proportion of each health worker’s salary. Mean relative time spent on each of the five categories of tasks was estimated per stockroom manager over the 2 days of participation, except one stockroom manager who only completed one ST. Stockroom managers’ relative time percentages were then combined and the mean, standard deviation, and 95% confidence interval were estimated for both CO and ST. Mean relative time for each category from the two methods were then compared using a paired *t* test. We repeated this analysis, calculating the relative distribution of the three productive categories within productive time and comparing between the CO and ST data.

Relative times calculated from CO and ST for each stockroom manager in each productive category were plotted on scatterplots to explore levels of consistency between the two methods. The scatterplots included a diagonal line that represents what would be seen if there was complete agreement between methods. Groupings of points close to the line represent higher consistency between the two methods, a dispersion of points shows low consistency, and groupings away from the line show consistent over- or underestimations by one method.

The cost of data collection for each method was calculated by adding up the vehicle mileage, gas costs, hourly wages of fieldworkers, and daily wages of the driver and dividing this total by the total number of observations attained for CO and the total number of timesheets filled out for ST.

## Results

### Characteristics of sample

The four health posts included in the study had a mean of 6.5 staff and provided a mean of 318 consultations monthly, 18.2% of which were FP consultations. The two health centres included in the study had a mean of 149.5 staff, including district-level health workers as the health centres shared sites with the health district, and provided a mean of 1 325 consultations per month, 17.8% of which were FP consultations (Table 1). COs were completed with all seven stockroom managers for 2 days; two STs were completed by all but one stockroom manager, who only completed one.

**Table 1:** Characteristics of health facilities included in TMS

Code	Job title	Facility Type	# of Staff	# of staff in supply provision	Average # of patients (monthly)*	Average # of FP consultations (monthly)*
HC1B	Centre Stockroom Manager	Centre	106	6	1 651	278
HP3	Post Stockroom Manager	Post	10	1	587	104
HP4	Post Stockroom Manager	Post	5	1	309	32
HC2A	Centre Supervising Stockroom Manager	Centre	193	6.5	1 000	193
HC2B	Centre Stockroom Assistant					
HP5	Post Stockroom Manager	Post	7	1	200	70
HP6	Post Stockroom Manager	Post	4	1	175	25

*Average of the whole calendar year

### Time use by stockroom managers

#### Continuous observation

Over the 12 days that the COs were conducted, 3 732 min (62 h 12 min) was observed across the seven stockroom managers, equivalent to an average of 4 h and 26 min per day. A mean of 4.1% (SD = 2.4%, CI = 2.8–5.5%) of time was spent on productive stock management activities associated with FP stock, and a mean of 47.0% (SD = 9.7%, CI = 41.7–52.3%) of time was spent on productive stock management activities for all other products. A mean of 9.5% (SD = 3.4%, CI = 7.6–11.3%) of time was spent on productive activities not related to stock management. A mean of 36.3% (SD = 11.9%, CI = 29.8–42.8%) of time was spent waiting for clients, and a mean of 3.2% (SD = 5.8%, CI = − 0.001 to 6.3%) of time was spent on non-productive activities (Table 2).

**Table 2:** Mean time estimates in absolute time and time percentage for Stockroom Managers from continuous observations and self-reported time sheets

Activity	Absolute time (H:MM)			Time percent (%)		
	Mean CO	Mean ST	Mean difference	Mean CO	Mean ST	Mean difference
FP productive stockroom activities	0:12	0:33	0:21	4.1%	9.8%	− 5.7%
Other productive stockroom activities	2:06	3:47	1:41	47.0%	65.7%	− 18.7%
Other productive activities	0:25	0:53	0:27	9.5%	15.1%	− 5.6%
Waiting time	1:32	0:13	1:19	36.3%	5.3%	31.0%
Non-productive	0:22	0:12	0:10	3.2%	4.1%	− 1.0%
Total	4:26	5:40	1:13	100%	100%	

#### Self-administered timesheets

During the 12 days of observation, stockroom managers completed 13 timesheets reporting 4 424 min (73 h 44 min) of daily activities. On average, stockroom managers reported activities totalling 5 h and 40 min per day. Stockroom managers reported spending on average 9.8% (SD = 3.7%, CI = 7.8–11.8%) of time on productive stock management activities dealing with FP, 65.7% (SD = 9.6%, CI = 60.4–70.9%) of time on productive stock management activities for all other products, 15.1% (SD = 8.0%, CI = 10.7%–19.5%) of time on productive activities unrelated to stock management, 5.3% (SD = 3.3%, CI = 3.5%–7.1%) of time waiting for clients, and 4.1% (SD = 1.5%, CI = 3.3%–4.9%) of time on non-productive activities (Table 2).

### Validation of the self-administered timesheet compared to continuous observation

**Fig. 2:**
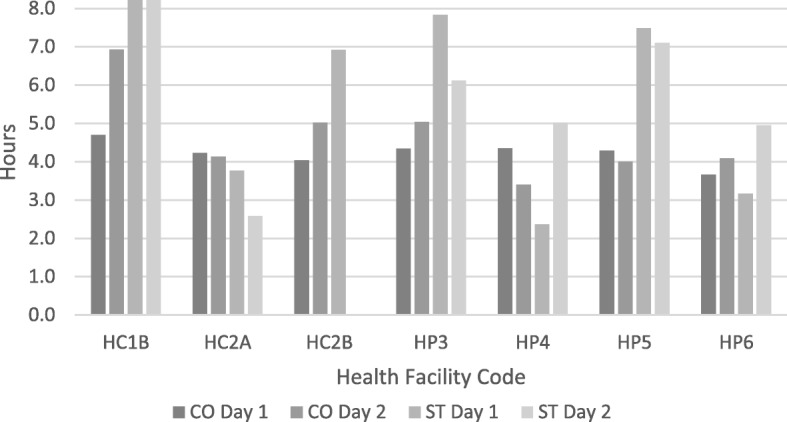
Comparison of total time recorded with CO and ST over 2 days in each facility

**Fig. 3:**
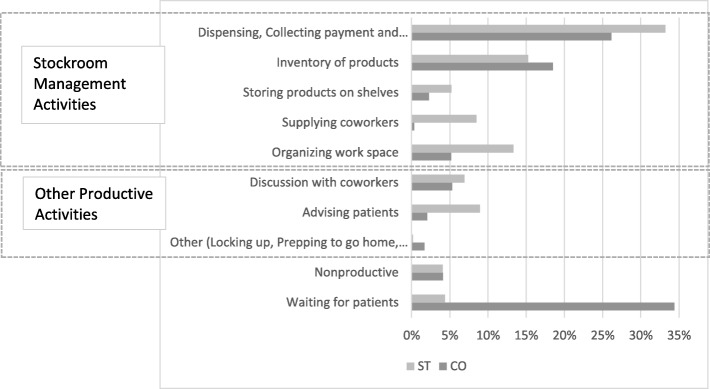
Comparison of relative time (%) allocation across activities measured by ST vs CO

Using ST, stockroom managers overestimated the length of their working day by a mean of 1 h and 20 min as compared to CO (Fig. 2), since it is difficult to make accurate time estimations. Thus, a large absolute time difference was found between CO and ST leading our analysis to examine relative time instead of absolute time. On the ST, stockroom managers overestimated the relative time spent for all three categories of productive activities: stock management activities associated with FP with a percent difference of 5.7% (95% CI 3.6–7.8%, *p* = 0.0005), stock management activities for non-FP products with a percent difference of 18.7% (95% CI 11.9–25.5%, *p* = 0.0004), and other productive activities with a difference of 5.6% (95% CI 0.5–11.0%, *p* = 0.08). Conversely, the stockroom managers underestimated time spent waiting for clients while filling out the ST by a percent difference of − 31.0% (95% CI − 38.4 to − 23.5%, *p* = 0.00001). On average, the stockroom managers accurately assessed the relative amount of non-productive time or breaks with just a 1.0% difference (95% CI − 1.9 to 3.8%, *p* = 0.56) (Fig. 3, Table 3).

**Table 3:** Statistical analysis of the mean difference of stockroom manager’s time between CO and ST for all categories (paired *t* test)

Stockroom managers’ relative time	Mean (μ)		Mean difference (MD)
	[95% CI]		*x̄*_CO_ − x̄_ST_ [95% CI]
All categories	CO	ST	ST − CO
FP productive stockroom activities	4.1%	9.8%	5.7% [3.6–7.8%]*
	[2.1–6.1%]	[7.3–12.3%]	
Other productive stockroom activities	47.0%	65.7%	18.7% [11.9–25.5%]*
	[38.9–55.1%]	[60.1–71.3%]	
Productive other	9.5%	15.1%	5.6% [0.4–10.9%]‡
	[6.5–12.4%]	[9.7–20.5%]	
Waiting time	36.3%	5.3%	− 31.0% [− 38.4 to − 23.5%]*
	[27.7–44.8%]	[1.5–9.1%]	
Non-productive	3.2%	4.1%	− 1.0% [− 1.9 to 3.8%]‡
	[0.6–5.7%]	[2.5–5.7%]	

*p < 0.001

‡*p* > 0.05

**Fig. 4:**
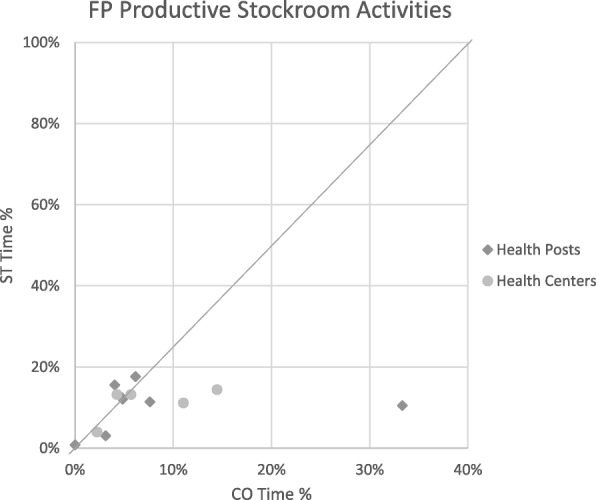
STs plotted against CO for productive stockroom activities time

**Fig. 5:**
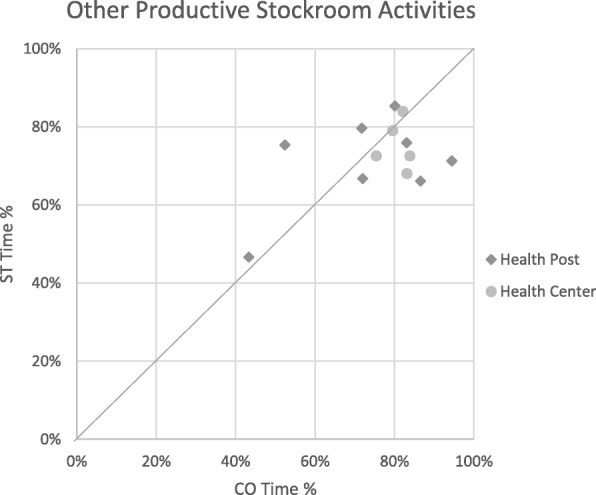
STs plotted against CO for other productive activities

**Fig. 6:**
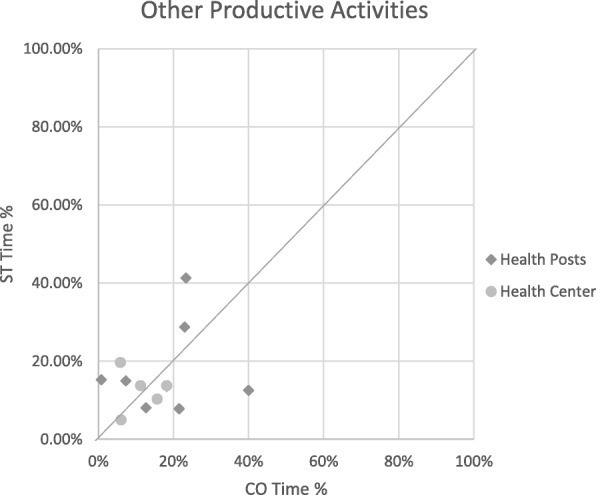
STs plotted against CO for non-productive activities

When comparing the relative distribution time spent on the three productive categories within productive time, the difference in time reported by ST relative to CO disappeared, with less than a 5% difference for all productive categories. The mean difference for time spent on the FP stockroom management activities became − 3.0% (95% CI − 7.4 to 1.4%; *p* = 0.25). For non-FP stockroom management activities, the mean percentage difference became 3.4% (95% CI − 2.8 to 9.6%; *p* = 0.35), and on other productive activities, it became − 0.1% (95% CI − 6.3 to 6.0%; *p* = 0.97), with ST estimates remaining higher than CO (Table 4). Scatterplots further explore the level of consistency of time percentage estimates between ST and CO for the three productive categories (Figs. 4, 5, and 6).

**Table 4:** Statistical analysis of the mean difference between CO and ST for productive categories only (paired *t* test)

Stockroom managers’ relative time	Mean (μ)		Mean difference (MD)
	[95% CI]		x̄_ST_ − x̄_CO_ [95% CI]
Productive categories	CO	ST	CO − ST
FP productive stockroom activities	7.8%	10.8%	− 3.0% [− 7.4 to 1.4%]‡
	[3.2–12.4%]	[8.04–13.5%]	
Other productive stockroom activities	76.0%	72.6%	3.4% [− 2.8 to 9.6%] ‡
	[68.4–83.6%]	[67.2–78.0%]	
Productive other	15.7%	16.8%	− 0.1% [− 6.3 to 6.0%] ‡
	[10.1–21.2%]	[10.5–21.1%]	

*p < 0.001

‡*p* > 0.05

### Cost of data collection

Data collection for CO cost $181 per observation day with a total of $1 424 for this study, compared to $59 per ST, totalling $558 (Table 5). This is due to the number of work hours required for the fieldworkers, paid hourly, for the different methods: the ST took 70 min on average to fill out and the CO lasted 5 h on average. As ST and CO data collection needed equal travel, and the driver was paid based on the trip distance, not the time spent waiting, the same driver and transport costs were applied to the two methods.

**Table 5:** Cost of data collection over 12 days

Role	# hours worked	Time (hours)^2^		Cost (USD)		Unit cost (USD)	
		CO	ST	CO	ST	CO (per day)	ST (per sheet)
Observer 1	60	50	10	$569.83	$113.96	$71.23	$14.25
Observer 2	54	45	9	$512.82	$102.56	$85.47	$20.51
Driver^1^	60	30	30	$341.50	$341.50	$24.39	$24.39
Total	174	125	49	$1 424.15	$558.02	$181.09	$59.15

^1^Includes driver’s daily rate, other transport costs, gas, and vehicle mileage

^2^Includes time spent travelling to and from health facilities from Dakar

## Discussion

This study used CO and ST methods of TMS to describe time use of stockroom managers working with a family planning supply chain intervention in Senegal. Out of an average of 2.7 h of productive time per day, stockroom managers spent most of this time on tasks directly related to stockroom management—with an average of 10% of that time going to FP products and 90% to all other products, followed by productive activities not associated with stockroom management—mostly discussions with co-workers. Only 4% of time was allocated to breaks, both scheduled and unscheduled. In comparing ST to CO, ST overestimates the absolute time spent working each day, particularly for productive tasks, and underestimates waiting time. In looking at absolute time or understanding productive time vs. unproductive time, STs do not give estimates consistent with CO. However, when non-productive and waiting time are removed from the total time, there was less than a 5% difference between the two methods in estimates of relative time spent on the three categories of productive time; this is half of the 10% cut off limit that was used to show consistency in a previous LMIC comparison study [7]. Finally, it was found that CO is a costlier data collection method than ST as it takes a whole shift and requires the presence of an external observer. In this pilot looking at 13 time periods, it cost $866 more, a rate which is 137.3% higher, to conduct the CO than the ST.

A variety of limitations were encountered in this study. This was a pilot study to develop tools for a larger study costing the FP supply chains in Senegal; due to resource constraints, the sample size was kept small. Similar time and motion studies in the United States of America and Australia also had small sample sizes of eight and nine nurses respectfully [7, 16]; however, the study in Ecuador compared 30 COs and STs [7]. Thus, our results are not fully generalizable to all stockroom managers across different facilities in Senegal due to purposive sampling. Additionally, our study took place during Ramadan when most of the stockroom managers, health workers, and community members were fasting. This led to smaller patient volumes and shorter workdays as staff went home early due to fatigue and low patient volume, thus making the absolute time results found unrepresentative for the whole year. It is possible that due to fatigue, hunger, or thirst, the health workers did not properly fill in the ST; however, our observers reported that all stockroom managers remained engaged during the process and did not seem too tired to fill out the sheet. Additionally, the objective of our study was to compare results from ST and CO, not to estimate the absolute amount of time spent on various activities, and we have no reason to believe that the comparison of ST and CO would be different at other times of the year or with a larger sample size.

Another limitation is that during the CO, stockroom managers may have changed their behaviour, a phenomenon well-described as the Hawthorne effect [19], leading to results showing more productive time. Stockroom managers were observed for 2 days to reduce any changes in behaviour and account for differences in patient volume on different days of the week. For the ST, desirability and recall bias may have led to overestimation of productive tasks, especially with FP stock management, as the stockroom managers knew the study was looking at IPM, which focuses on the FP supply chain management, and most people want to look productive on paper, even if anonymous. As the Hawthorne effect of CO, and desirability and recall biases of ST all lead to overestimation of productive time, it is unlikely that these will have affected the overall comparison between CO and ST.

The limitations of self-reporting methods like STs are well-documented and describe how self-reporting overestimates productive time and underestimates non-productive time partially due to the desirability bias [7, 10] and partially because it is difficult to quantify more abstract tasks, like administration [7]. In our study, we found that although the absolute time and relative time spent on productive activities were overestimated by respondents in STs, the relative distribution within those productive activities was not overestimated for stockroom management tasks. Other studies also found that using percentage time in the LMIC context allowed for accurate representation of time costs for larger costing studies and facilitated the calculations of monthly and yearly personnel costs [7, 16]. Since our CO and ST results differed by less than 5% in the relative distribution of productive activities, it may be feasible to create an adjustment factor for future STs completed in Senegal based on the percentage mean difference between CO and ST results in this study.

In contrast to the studies in high-income countries that compare CO and ST, our study shows consistency between methods; this agrees with the study that compared TMS methods in Ecuador [7], finding less than 10% difference in results from CO and ST methods. The Australian TMS study concluded that ST is not valid compared to CO, even though there was less than 10% difference in findings between the two methods; however, compliance rates for the ST were low at only 56% [18]. We had a much higher compliance rate with our surveys at 92.8% since our fieldworkers were present while the STs were filled out. This suggests that to ensure STs are representative of productive relative time in future studies, steps need to be taken to ensure high compliance. The study in the United States of America found that perceptual differences between participants regarding task allocation made the STs less accurate [17]; our study addressed this issue by limiting the choice of work activities on our timesheet and by having a researcher present for questions on task allocation.

Similar to other TMS in LMICs we found that time spent waiting for clients was underestimated in ST (4%) relative to CO (34%). Our CO relative time percentages compare to the 29.6% of time health extension workers spent waiting in a CO in Ethiopia and the 43% of time community nurses in Tanzania spent waiting for clients, as observed in another CO [7, 8, 13]. The stockroom managers in our study are not paid for the time they spend waiting for patients or on non-productive tasks and there may not currently be enough demand for medications or other medical products in these health facilities to justify their full day of work. However, the stockroom managers are the only staff who can dispense medications in most health facilities in Senegal, so if they do not work the same hours as the nurses and midwifes, people would not be able to get their needed medications. This is especially problematic with FP, as women in Senegal stated that closed stockrooms are a barrier to continuing contraceptive use [20]. Including non-productive and waiting time as separate categories in our TMS is valuable for determining potential productivity gains in LMICs [21] or for determining shorter or longer work schedules for stockroom managers. Waiting time estimates might be better used to identify areas for task shifting, allowing for the stockroom managers to become involved in nonspecialized tasks in reproductive health services, such as administering injectable FP, as studies in Sub-Saharan Africa are examining [22, 23]. In Senegal, head nurses, clinic managers, and midwifes at the health facility level have reported spending large amounts of time reporting to the districts, so the feasibility of shifting these tasks to stockroom managers needs to be explored. If stock managers received the extra training and income that is not solely based on sales, such task-shifting could allow the intended goal of IPM-3PL to be realized, namely allowing for time-savings to be used to increase direct provision of FP services. As the Senegalese government is currently focused on FP services and improved supply chains, it is an opportune time to understand how to capitalize on stockroom managers’ unproductive time.

## Conclusion

In conclusion, we have demonstrated that alone, STs are not a reliable method to collect data on absolute labour time or for comparing non-productive versus productive activities. However, while COs provide more reliable time estimates, the unit costs for data collection are more than triple those of ST in Senegal. Since the mean differences in relative time estimates are less than 5% when analysing categories within productive activities, the extra cost of CO needs to be considered where limited data collection resources are available for studies interested in the distribution of productive activities. Future studies that plan to calculate personnel time should incorporate a pilot study, to compare differences in CO and ST, and understand the difference in mean times collected by the two methods to appropriately weigh any results for a given context.

## Abbreviations

CO: Continuous observation
FP: Family planning
IPM-3PL: Informed push model with 3rd party logisticians
LMIC: Low- and middle-income country
PI: Provider interview
ST: Self-administered timesheet
TMS: Time and motion study

## References

[1] Daff BM, Seck C, Belkhayat H, Sutton P (2014). Informed push distribution of contraceptives in Senegal reduces stockouts and improves quality of family planning services. Glob Health Sci Pract.

[2] 2011 Baseline Survey for the Senegal Urban Health Initiative (ISSU) Service Delivery Site Survey. Final Report Dakar: IntraHealth International. URHI; 2012. https://docplayer.net/30571141-2011-baseline-survey-for-the-senegal-urban-health-initiative-issu-service-delivery-site-survey-final-report-april-2012.html. Accessed 20 Jan 2017.

[3] Shretta R, Johnson B, Smith L, Doumbia S, de Savigny D, Anupindi R (2015). Costing the supply chain for delivery of ACT and RDTs in the public sector in Benin and Kenya. Malar J.

[4] Baruwa E, Tien M, Sarley D. Zambia ARV Supply chain costs: a pilot of the supply chain costing tool. Arlington: U SAID, 2010. http://gestionensalud.medicina.unmsm.edu.pe/wp-content/uploads/2015/08/CM_RB_03_ZM_ARVSupplyChainCost.pdf. Accessed 25 Jan 2017.

[5] Sarley D, Baruwa E, Tien M. Zimbabwe: supply chain costing of health commodities. Arlington: USAID, 2010. https://www.jsi.com/JSIInternet/Resources/publication/display.cfm?txtGeoArea=INTL&id=12237&thisSection=Resources. Accessed 25 Jan 2017.

[6] Routh S, Thwin AA, Barb N, Begum A (2004). Cost efficiency in maternal and child health and family planning service delivery in Bangladesh: implications for NGOs. Health Policy Plan.

[7] Bratt JH, Foreit J, Chen PL, West C, Janowitz B, de Vargas T (1999). A comparison of four approaches for measuring clinician time use. Health Policy Plan.

[8] Manzi F, Schellenberg JA, Hutton G, Wyss K, Mbuya C, Shirima K (2012). Human resources for health care delivery in Tanzania: a multifaceted problem. Hum Resour Health.

[9] Adam T, Amorim DG, Edwards SJ, Amaral J, Evans DB (2005). Capacity constraints to the adoption of new interventions: consultation time and the integrated management of childhood illness in Brazil. Health Policy Plan.

[10] Mangham-Jefferies L, Mathewos B, Russell J, Bekele A (2014). How do health extension workers in Ethiopia allocate their time?. Hum Resour Health.

[11] Were MC, Sutherland JM, Bwana M, Ssali J, Emenyonu N, Tierney WM (2008). Patterns of care in two HIV continuity clinics in Uganda, Africa: a time-motion study. AIDS Care.

[12] Lopetegui M, Yen P-Y, Lai A, Jeffries J, Embi P, Payne P (2014). Time motion studies in healthcare: what are we talking about?. J Biomed Inform.

[13] Tilahun H, Fekadu B, Abdisa H, Canavan M, Linnander E, Bradley EH (2017). Ethiopia’s health extension workers use of work time on duty: time and motion study. Health Policy Plan.

[14] Wirth P, Kahn L, Perkoff GT (1977). Comparability of two methods of time and motion study used in a clinical setting: work sampling and continuous observation. Med Care.

[15] Finkler SA, Knickman JR, Hendrickson G, Lipkin M, Thompson WG (1993). A comparison of work-sampling and time-and-motion techniques for studies in health services research. Health Serv Res.

[16] Bonenberger M, Aikins M, Akweongo P, Bosch-Capblanch X, Wyss K (2015). What do district health managers in Ghana use their working time for? A case study of three districts. PLoS One.

[17] Burke TA, McKee JR, Wilson HC, Donahue RMJ, Batenhorst AS, Pathak DS (2000). A comparison of time-and-motion and self-reporting methods of work measurement. J Nurs Adm.

[18] Ampt A, Westbrook J, Creswick N, Mallock N (2007). A comparison of self-reported and observational work sampling techniques for measuring time in nursing tasks. J Health Serv Res Policy.

[19] Fernald DH, Coombs L, DeAlleaume L, West D, Parnes B (2012). An assessment of the Hawthorne effect in practice-based research. J Am Board Fam Med.

[20] Cavallaro FL, Duclos D, Faye S, Faye A, Cresswell J, Lynch C (2017). Understanding “missed appointments” for women’s refills of pills and injectables in Senegal: a mixed methods study. IUSSP; 29 October - 4 November; Capetown, SA.

[21] Kurowski C, Wyss K, Abdulla S, Yémadji ND, Mills A. Human resources for health: requirements and availability in the context of scaling-up priority interventions in low-income countries. Global Resour Center 2003. https://www.hrhresourcecenter.org/node/2700.html. Accessed 21 July 2017.

[22] Naburi H, Ekström AM, Mujinja P, Kilewo C, Manji K, Biberfeld G (2017). The potential of task-shifting in scaling up services for prevention of mother-to-child transmission of HIV: a time and motion study in Dar es Salaam, Tanzania. Hum Resour Health.

[23] Chin-Quee D, Bratt J, Malkin M, Nduna MM, Otterness C, Jumbe L (2013). Building on safety, feasibility, and acceptability: the impact and cost of community health worker provision of injectable contraception. Glob Health: Sci Pract.

